# Identifying Candidate Genes for Enhancing Grain Zn Concentration in Wheat

**DOI:** 10.3389/fpls.2018.01313

**Published:** 2018-09-10

**Authors:** Dalia Z. Alomari, Kai Eggert, Nicolaus von Wirén, Ahmad M. Alqudah, Andreas Polley, Jörg Plieske, Martin W. Ganal, Klaus Pillen, Marion S. Röder

**Affiliations:** ^1^Leibniz Institute of Plant Genetics and Crop Plant Research (IPK), Gatersleben, Germany; ^2^SGS TraitGenetics GmbH, Gatersleben, Germany; ^3^Institute of Agricultural and Nutritional Sciences, Martin-Luther-University Halle-Wittenberg, Halle, Germany

**Keywords:** Zinc, *Triticum aestivum*, wheat quality, micronutrient, GWAS

## Abstract

Wheat (*Triticum aestivum* L.) is one of the major staple food crops worldwide. Despite efforts in improving wheat quality, micronutrient levels are still below the optimal range for human nutrition. In particular, zinc (Zn) deficiency is a widespread problem in human nutrition in countries relying mainly on a cereal diet; hence improving Zn accumulation in grains is an imperative need. This study was designed to understand the genetic architecture of Zn grain concentrations in wheat grains. We performed a genome-wide association study (GWAS) for grain Zn concentrations in 369 European wheat genotypes, using field data from 3 years. The complete wheat panel was genotyped by high-density arrays of single nucleotide polymorphic (SNP) markers (90k iSELECT Infinium and 35k Affymetrix arrays) resulting in 15,523 polymorphic markers. Additionally, a subpanel of 183 genotypes was analyzed with a novel 135k Affymetrix marker array resulting in 28,710 polymorphic SNPs for high-resolution mapping of the potential genomic regions. The mean grain Zn concentration of the genotypes ranged from 25.05–52.67 μg g^-1^ dry weight across years with a moderate heritability value. Notably, 40 marker-trait associations (MTAs) were detected in the complete panel of varieties on chromosomes 2A, 3A, 3B, 4A, 4D, 5A, 5B, 5D, 6D, 7A, 7B, and 7D. The number of MTAs in the subpanel was increased to 161 MTAs whereas the most significant and consistent associations were located on chromosomes 3B (723,504,241–723,611,488 bp) and 5A (462,763,758–466,582,184 bp) having major effects. These genomic regions include newly identified putative candidate genes, which are related to Zn uptake and transport or represent bZIP and mitogen-activated protein kinase genes. These findings provide the basis for understanding the genetic background of Zn concentration in wheat grains that in turn may help breeders to select high Zn-containing genotypes to improve human health and grain quality.

## Introduction

Wheat is among the primary staple crops in the world and its production reached almost 750 million tons per year (FAOSTAT, 2016^1^), while 68% of the yield is used for human nutrition (FAOSTAT, 2012). Wheat provides substantial amounts of mineral elements, which are beneficial for human health. Several reports emphasize that over 2 billion of people are suffering from hidden hunger (Welch and Graham, 2004), i.e., Zinc (Zn) and Iron (Fe) deficiency, mainly in middle- or low-income countries where staple crops are the major food source (Sands et al., 2009); recently, the problem was also reported in developed countries (Pandey et al., 2016).

Zn plays significant roles in different metabolic processes and is an essential cofactor for many enzymes and regulatory proteins. The symptoms of insufficient dietary Zn intake for humans can be observed as growth and development retardation, excessive weight loss, diarrhea, and depression (Ozturk et al., 2006; Kambe et al., 2014; Krishnappa et al., 2017). Consequently, improving the nutritional quality of wheat grains by enhancing Zn concentrations is a long-term goal for breeding novel wheat cultivars with a positive effect on grain yield, nutritional quality of the plant, as well as human health (Cakmak, 2008; Genc et al., 2008; Crespo-Herrera et al., 2016).

Since Zn accumulation in grains is a genetically complex trait, genome-wide association study (GWAS) is a powerful tool to detect the genetic factors underlying the natural variation in such complex traits (Hamblin et al., 2011). Several studies identified quantitative trait loci (QTL) for micronutrients, such as Fe and Zn, or macronutrients like Ca in wheat (Morgounov et al., 2007; Tiwari et al., 2009; Crespo-Herrera et al., 2016; Alomari et al., 2017). Peleg et al. (2009) found six QTLs on chromosomes 2A, 2B, 3A, 4B, 5A, 6A, 6B, 7A, and 7B for Zn in a durum wheat × emmer wheat recombinant inbred lines (RILs) population. Four QTLs for grain Zn concentration were identified by Genc et al. (2008) on chromosomes 3D, 4B, 6B, and 7A in a doubled haploid wheat population. Another study mentioned seven QTLs located on chromosomes 1A, 2D, 3A, 4A, 4D, 5A, and 7A for Zn content in wheat grains of which four QTLs are shared with Zn concentration (Shi et al., 2008). Shi et al. (2013) found that chromosome 4D and 5A probably very vital in controlling mineral status in wheat grains.

Previous studies on Zn concentration mainly used bi-parental population, for instance, RIL (Xu et al., 2012; Pu et al., 2014; Srinivasa et al., 2014) but a few studies have used GWAS with high dense single nucleotide polymorphic (SNP) arrays to investigate the genomic regions underlying the accumulation of micronutrients including Zn in the grains of major cereals like wheat (Guttieri et al., 2015). Therefore, understanding the genetic background of Zn accumulation in wheat grains by GWAS provides the basis for devising the plant breeding strategies and for improving the grain Zn status by introducing the putative candidate genes based on the newly available wheat reference (IWGSC RefSeq v1.0) and using advanced bioinformatics tools.

The main goals of this study were (i) to investigate the natural phenotypic variation on grain Zn concentrations for 369 wheat varieties of 3 years field experiments, (ii) to study the genetic architecture of Zn grain concentration by GWAS analysis with three different high dense SNP arrays including 44,233 SNPs providing a high-resolution genetic map, and (iii) to identify the genomic regions and potential candidate genes for consistently significant QTLs.

## Materials and Methods

### Plant Material and Field Trials

In this study, we used 369 European elite wheat varieties including 355 genotypes of winter wheat and 14 spring wheat genotypes, originating from Germany, France, Poland, Denmark, Austria, Czech Republic, United Kingdom, Sweden, Switzerland, Hungary, Italy, Belgium, and Netherlands described in (Kollers et al., 2013). Field trials were conducted at IPK, Gatersleben, Germany within 3 years (2014/2015 for 358 genotypes, 2015/2016 for 365 genotypes, and 2016/2017 for 360 genotypes). Few genotypes were missing in each individual year due to poor performance and loss in the field. Each plot size was 2 m × 2 m with six rows spaced 0.20 m apart. Plants were grown in clayey loam soil with phosphorus ranges between 7.1–9.0 μg g^-1^ and pH ≈ 7 across years. Standard agronomic wheat management practices were applied without using fertilizers to avoid the effect of additional fertilizers on the actual Zn concentrations.

### Wheat Grain Samples Preparation and Milling

The complete panel of genotypes was analyzed for each individual year. For each genotype, thousand kernel weight (TKW) was measured using a digital seed analyzer/counter Marvin (GTA Sensorik GmbH, Neubrandenburg, Germany). Grains were milled using a Retsch mill (MM300, Germany) and the complete panel of the milled samples was dried by incubating overnight at 40°C.

### Measuring Grain Zinc Concentration

Fifty milligrams of dried and milled wheat grain flour was taken to be digested by (2 ml) nitric acid (HNO_3_ 69%, Bernd Kraft GmbH, Germany). The digestion process was performed using a high-performance microwave reactor (UltraClave IV, MLS, Germany). All digested samples were filled up to 15 ml final volume with de-ionized distilled (Milli-Q^®^) water (Milli-Q Reference System, Merck, Germany). Element standards were prepared from Bernd Kraft multi-element standard solution (Germany). Zinc as an external standard and yttrium (Y) (ICP Standard Certipur^®^ Merck Germany) were used as internal standards for matrix correction. Zinc concentrations were measured by inductively coupled plasma optical emission spectrometry (ICP-OES, iCAP 6000, Thermo Fisher Scientific, Germany) combined with a CETAC ASXPRESS^TM^ PLUS rapid sample introduction system and a CETAC autosampler (CETAC Technologies, Omaha, NE, United States).

### Statistical Analysis

The broad-sense heritability (H^2^) was calculated using the equation:

(1) $$H^{2}=\;\sigma_{G}^{2}/(\sigma_{G}^{2}\;+\;(\sigma_{e}^{2}/nE)$$

where $\sigma_{G}^{2}$ is the variance of the genotype, $\sigma_{e}^{2}$ represents the variance of the residual, and nE is the number of the environments.

Analyses of variance (ANOVA) and Pearson’s correlation coefficient were calculated for the grain Zn trait across 3 years with Sigma Plot package 13.

Best linear unbiased estimates (BLUEs) based on mixed linear models (MLMs) function with applying the residual maximum likelihood (REML) algorithm were calculated to analyze the phenotypic data and estimate the mean of each individual over the years (Yu et al., 2006). To this end, the genotype term was considered as a fixed effect and we denote year as environment term, which was considered as a random effect. These calculations were accomplished using GenStat v16 software (VSN International, Hemel Hempstead, Hertfordshire, United Kingdom).

### SNP Genotyping and GWAS Analysis

The complete wheat panel consisting of 369 varieties was genotyped by TraitGenetics GmbH, Gatersleben, Germany^2^ using two marker arrays: a 90k iSELECT Infinium array (Wang et al., 2014) and a 35k Affymetrix-SNP array (Axiom^®^ Wheat Breeder’s Genotyping Array^3^; Allen et al., 2017). Additionally, a novel 135k Affymetrix array designed by TraitGenetics was used to genotype a subpanel of 183 genotypes from the complete panel of genotypes (Zanke et al., 2017). For the reference map, the ITMI-DH population (Sorrells et al., 2011; Poland et al., 2012) was used to anchor the SNP-markers of the 90k and 35k arrays. The 135k array markers were genetically mapped on four different F_2_-populations and then physically anchored on the reference sequence RefSeq v1.0 of hexaploid wheat^4^ from International Wheat Genome Sequencing Consortium (IWGSC). For SNP markers quality control, we applied a minor allele frequency (MAF) ≤ 3% (equaling 11 varieties out of 369) with rejecting SNPs having missing values or heterozygosity ≥ 3%, resulting in 7,761 mapped polymorphic SNP markers from the 90k iSELECT, 7,762 SNPs from the 35k Affymetrix-SNP, and 28,710 from the 135k Affymetrix, which were used for association analysis. The investigated genotype panel and its population structure were described in a previous study by Kollers et al. (2013).

Association mapping based on a MLM was conducted primarily using the Genome Association and Prediction Integrated Tool (GAPIT; Lipka et al., 2012) in R: a language and environment for statistical computing. It includes the phenotypic data with SNP markers coming from the high-density arrays. We incorporated PCA for population correction and stratification. For significant marker-trait associations (MTAs) detection, we set a threshold P-value of -log_10_ (*P*) ≥ 3. Quantile-quantile plots were drawn based on the observed and expected -log_10_ (*P*) values. Explained phenotypic variance (*R*^2^) and marker effects (positive/negative) were extracted from GWAS results.

### Connecting Significant SNPs With the Physical Sequence of Wheat

The flanking sequence of significant SNP markers defining significant associations with the grain Zn concentration trait was obtained from the wheat 90k database (Wang et al., 2014), 35k database^5^ and 135k Affymetrix array (unpublished data, TraitGenetics). These flanking sequences were blasted by Galaxy software, which is an IPK-internal web-based platform^6^ by using megablast to fetch the whole sequence of the genomic region of interest based on IWGSC RefSeq v1.0. The extracted sequences were submitted to the annotation pipeline MEGANTE^7^ in order to identify potential candidate genes and their gene ontologies.

## Results

### Natural Phenotypic Variation of Grain Zn Concentrations in Two Wheat Panels

**FIGURE 1:**
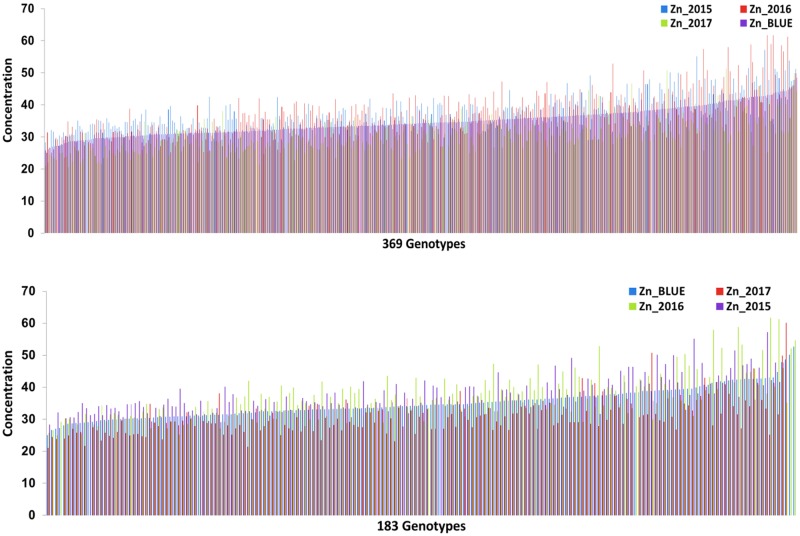
**(A)** Zn concentration distribution for the whole wheat genotypes panel (369) in the 3 years (2015/2016/2017) and BLUE. **(B)** Zinc concentration distribution for the subpanel of wheat genotypes (183) in the 3 years (2015/2016/2017) and BLUE.

**FIGURE 2:**
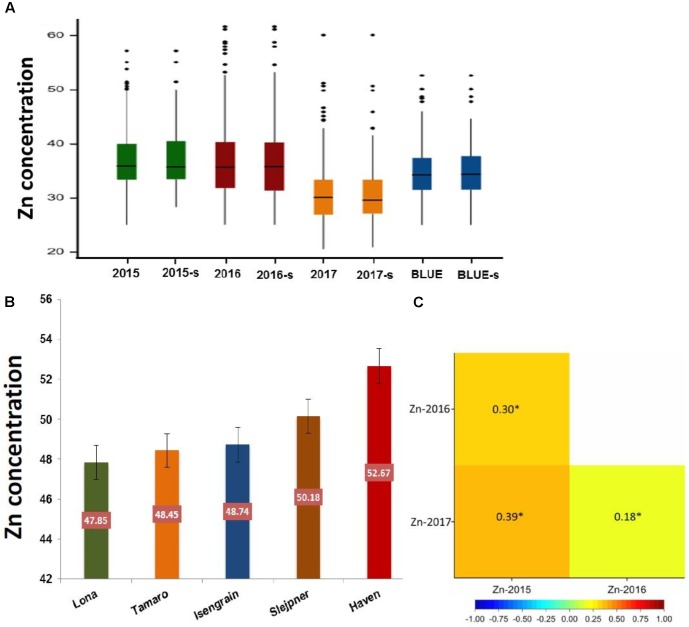
**(A)** The boxplots of Zn concentration for the whole panel (369 genotypes) and a subpanel (183 genotypes) among 3 years and BLUE. **(B)** The scale of the top five genotypes with the highest Zn concentration value based on BLUE values. **(C)** Person correlation between years.

Zn measurements were obtained from grain samples of 369 European wheat varieties, which were grown under field conditions in three consecutive years (2015, 2016, and 2017). Zn concentrations of each individual wheat genotype for the complete panel of 369 genotypes and for the subpanel with 183 genotypes are presented in **Supplementary Table S1**. The phenotypic distribution of the Zn concentrations in the individual years appeared to be normally distributed (**Supplementary Figure S1**). A wide range of variation in the Zn concentration was observed for the complete panel (**Figure 1A**) and the subpanel (**Figure 1B**) in all 3 years and most of the variation within the complete panel was also captured in the subpanel (**Figure 2A** and **Table 1**). The results of BLUEs across 3 years’ data ranged from 25.05 to 52.67 μg g^-1^DW with a mean of 34.92 μg g^-1^ DW. The genotype “Haven” had the highest Zn concentration equaling 52.67 μg g^-1^ DW in the complete panel of wheat grain genotypes based on the BLUEs (**Figure 2B**). A significant positive Pearson’s correlation ranging from *r* = 0.18 to 0.39 (*P* < 0.001) among the years (**Figure 2C**) indicated a relatively stable measurement of the phenotypes. A significant positive Pearson’s correlation was found between Zn and TKW in all 3 years (**Supplementary Figure S2**). The broad-sense heritability for Zn concentration across the years was *H*^2^ = 0.54. The results of ANOVA for Zn concentration indicated significant effects of genotype and environment, i.e., years (**Supplementary Table S2**).

**Table 1:** Grain Zn concentration mean, median, minimum, and maximum values within the complete and subpanel of wheat genotypes for each individual year.

Year-panel	Observation number	Mean μg g^-1^	Median μg g^-1^	Minimum μg g^-1^	Maximum μg g^-1^
2015-Complete panel	358	37.10	35.90	25.00	57.17
2015-Subpanel	176	37.34	35.75	28.34	57.17
2016-Complete panel	365	36.92	35.70	25.10	61.70
2016-Subpanel	180	36.83	35.80	25.10	61.70
2017-Complete panel	359	30.61	30.12	20.56	60.09
2017-Subpanel	176	30.80	29.64	21.00	60.09


### Association Mapping of Grain Zn Concentrations in Two Diverse Wheat Panels

**FIGURE 3:**
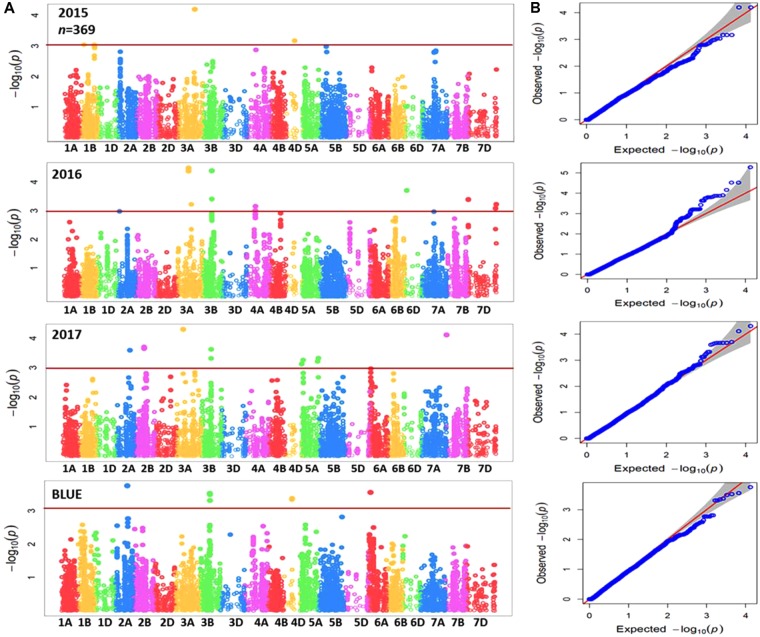
**(A)** Summary of genome-wide association plots output of Zn concentration for the whole panel of wheat genotypes (369) which analyzed by using 90k and35k for each year (2015/2016/2017) and BLUEs with mixed linear model and PCA model. **(B)** Quantile-quantile scale representing expected versus observed *P*-values at -log10 (*P*).

**FIGURE 4:**
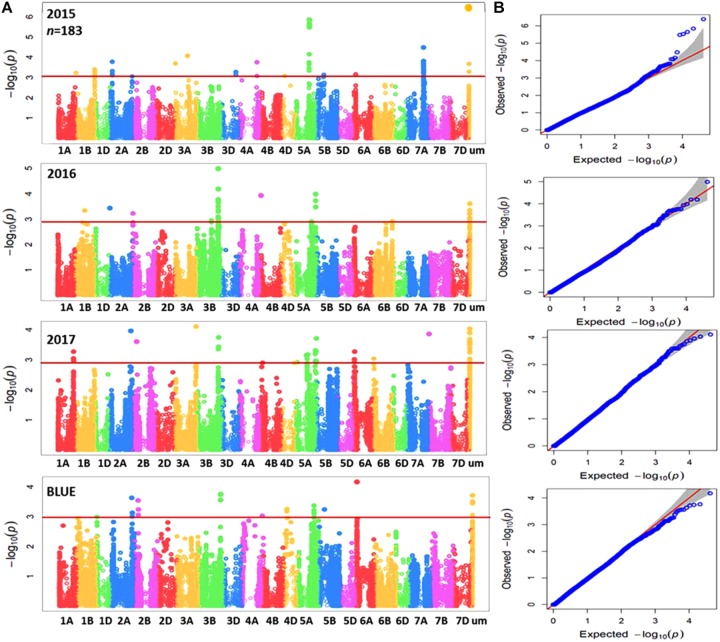
**(A)** Summary of genome-wide association plots output of Zn concentration for the subpanel of wheat genotypes (183) which analyzed by 90k, 35k, and 135k for each year (2015/2016/2017) and BLUEs using mixed linear model and PCA model. **(B)** Quantile-quantile scale representing expected versus observed *P*-values at -log10 (*P*).

Genome-wide association mapping was performed for the complete panel and subpanel of wheat genotypes with Zn concentration data for each individual year in addition to BLUEs, using the implemented MLM with applying principal component analysis (PCA) as a correction factor for population structure. The complete panel of wheat genotypes was analyzed by a combination of markers from the 90K iSELECT INFINIUM array and the 35K Affymetrix array resulting in 15,523 polymorphic SNP markers which were anchored in a genetic reference map. The subpanel was analyzed by merging 90K iSELECT array, 35K and 135k Affymetrix arrays resulting in a total of 44,233 polymorphic SNP markers based on their physical locations in order to increase the density of markers, achieve good mapping resolution, and to further enhance the power of GWAS output within the germplasm panel. Significant MTAs were detected above the threshold of –log10 (*P*-value) ≥ 3 as shown in Manhattan plots for both panels (**Figures 3A, 4A**). The GWAS results were presented along with the QQ plots for SNPs, revealing that the distributions of observed association *P*-values were close to the distribution of expected associations (**Figures 3B, 4B**). A total of 40 MTAs were detected in the complete panel on chromosomes 2A, 3A, 3B, 4A, 4D, 5A, 5B, 5D, 6D, 7A, 7B, and 7D with *R*^2^-values ranging from 2.5 to 5.2%. A total of 21 MTAs had positive effects related to the minor allele and 19 MTAs had negative effects (**Supplementary Table S3**). While most MTAs were only detected in 1 year, an MTA on chromosome 3B was detected in all 3 years in similar mapping locations of 64.5 to 66.8 cM. The most significant MTA was detected on chromosome 5A with -log (*p*) value equaling 4.87 in the genomic region of 114.5 cM and explaining an *R*^2^ value of 5.2%. The number of MTAs in the subpanel was increased to 161 including 31 unmapped markers on chromosomes 1A, 1B, 2A, 2B, 3A, 3B, 3D, 4A, 4D, 5A, 5B, 6A, 6B, 7A, and 7B with *R*^2^-values ranging from 5.5 to 13.7% (**Supplementary Table S4**). A genomic region on chromosome 3B between the physical location of 716,993,339 and 736,712,355 (IWGSC RefSeq v1.0) is defined by 26 MTAs in the years 2016, 2017 and BLUEs with the highest *R*^2^ of 11.3% at AX-95129199. A continuous range of 27 significant MTAs was detected on chromosome 5A ranging from physical location 353,989,023–698,510,016 including all 3 years and BLUEs. The most significant marker AX-158550766 located at position 464,479,275 explained 12.3% of phenotypic variation. A total of six markers for chromosome 3B (64.5–66.8 cM) and two markers for chromosome 5A (98.1–114.5 cM) were shared between the complete panel of varieties and the subpanel.

### Defining Physical Regions of Candidate Genes Underlying Zn Accumulation in Wheat Grains

**FIGURE 5:**
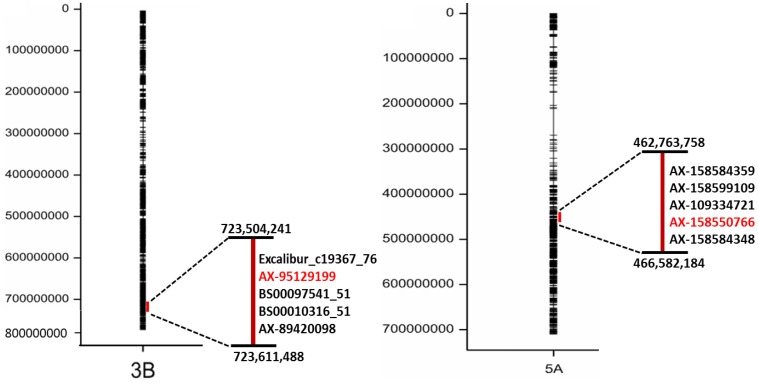
Alignment of the significant SNP markers (in black) to chromosome 3BS and 5AL.The most significant SNP (in red) with –log *(P)* value equaling 5.84.

The highly significant SNP markers that located on chromosome 3B and 5A (**Figure 5**) were selected for BLAST analysis, using the web-based platform Galaxy^6^. The physical region of these SNPs at chromosome 3B located between 723,504,241 to 723,611,488 bp and for 5A on 462,763,758 to 466,582,184 bp (**Figure 5**) that were queried against IWGSC RefSeq v1.0. The fetched sequence output from Galaxy was submitted to MEGANTE^7^, which is a web-based system for integrated plant genome annotation to perform genome annotations. On chromosomes 3B and 5A, we found a number of genes encoding proteins with known functions and others reported as hypothetical proteins (**Supplementary Table S5**). Putative candidate genes based on their function included a transcription factor (TF) belonging to the basic leucine zipper (bZIP) family and the TF bHLH76, a homeobox-leucine zipper protein HOX4, a SWAP (suppressor-of-white-apricot)/surp domain-containing protein and several genes related to the mitogen-activated protein kinase (MAPK) gene family (**Table 2**). Thus, we conclude that these two genomic regions on chromosomes 3B and 5A harbor a number of putative candidate genes, which may have a significant role in the process of grain Zn accumulation.

**Table 2:** Summary of putative candidate genes associated with Zn grain concentration in wheat.

Gene number	Description	GO ID	GO term	GO category	Chr.
mRNA_2.1	Mitogen- activated	GO:0004672	Protein kinase activity,	Molecular	3B
mRNA_3.1	protein kinase kinase	GO:0006468	protein phosphorylation,	function,	
mRNA_10.1	kinase	GO:0015031	protein transport	biological	
mRNA_23.1				process	
mRNA_24.1					
mRNA_32.1	SWAP (suppressor-of-white-APRICOT)/surp domain-containing protein	GO:0003723	RNA binding	Molecular function	3B
mRNA_11.1	Homeobox-leucine zipper protein HOX4	GO:0003677/GO:0003700	DNA binding/DNA binding transcription factor activity	Molecular function	5A
mRNA_34.1	Protein FAR1-RELATED SEQUENCE 11 (FRS11)	GO:0008270	Zinc ion binding	Molecular function	5A
mRNA_42.1	BZIP protein	GO:0003700	DNA binding transcription factor activity	Molecular function	5A
mRNA_44.1	Transcription factor bHLH76	GO:0046983	Protein dimerization activity	Molecular function	5A


## Discussion

### Wide Variation for Zn Accumulation in Wheat Grains

The poor bioavailability of essential nutrients in cereal grains leads the breeders to use plant breeding which is a seed-based approach to develop cultivars with improved and adequate levels of nutrients (Tiwari et al., 2016). Plant breeding or genetic biofortification was found to be comparative with other costly and non-sustainable approaches such as agronomic biofortification which is based on using fertilizers or other approaches that are based on food fortification and daily consumed supplementations (Garcia-Oliveira et al., 2018). Therefore, genetic biofortification is considered as one of the vital

approaches that can help to overcome malnutrition problems either by classical plant breeding or approaches involving GMOs (genetically modified organisms) (Borrill et al., 2014; Singh et al., 2017). Many reports mentioned that the targeted range for biofortified grains and to develop cultivars with high Zn concentration is between 40–50 μg g^-1^ (Howarth et al., 2011; Cakmak and Kutman, 2017). The phenotypic variation that found in our germplasm ranged from 25.05–52.65 μg g^-1^ which is compatible with the target range and provides the chance to use the highest grain Zn-containing genotypes in breeding programs. Similar Zn concentration ranges were also reported by Graham et al. (1999) and Guttieri et al. (2015) who found that Zn concentrations in 132 bread wheat genotypes ranged between 25–53 and 13.1–45.2 μg g^-1^ in hexaploid wheat. Additionally, in durum wheat, the variation ranged from 24.8–48.8 μg g^-1^ for Zn which is comparable with our observations (Magallanes-Lopez et al., 2017).

Grain Zn concentrations across years were weakly to moderately correlated (*r* = 0.18–0.39; *P* < 0.001) which may be attributed to environmental effects across years and its interaction with genotypes as was also reported for grains of other crops (Singh et al., 2017). The calculated heritability (*H*^2^ = 0.54) for our trait of interest represents a moderate contribution of the genotype to the overall variation in grain Zn concentration, which was also affected by the environment in experiments being conducted across 3 years. Similarly, Tiwari et al. (2016) and Khokhar et al. (2018) found the moderate effect of genotypes on wheat grain Zn concentrations. We observed a significant positive correlation between Zn and TKW, which implies that both traits improve simultaneously each other and this observation has also been made in other studies with wheat (Morgounov et al., 2007; Peleg et al., 2009; Krishnappa et al., 2017). Our findings provide a list of improved cultivars with high Zn concentration that can be utilized in future breeding programs for boosting grain quality.

### Zn Grain Concentration as a Complex Trait

Genetic dissection for grain Zn concentration in our diversity panel showed that this trait is under control of many genetic loci. The constant significant MTAs across the years 2015, 2016, 2017 and BLUE values are conferred by loci on chromosomes 3B (723,504,241–723,611,488 bp) and 5A (462,763,758–466,582,184 bp) in the complete panel as well as in the subpanel of wheat genotypes (**Figure 5**). Previously, a QTL for Zn concentration was reported in a similar location on chromosome 3B by Crespo-Herrera et al. (2017) in a population of hexaploid wheat RILs. Another study detected on chromosome 5A a QTL for grain Zn concentration in a RIL population derived from a cross between durum wheat and wild emmer wheat (Peleg et al., 2009). A previous study in rice mentioned that many QTLs for grain Zn have been mapped based on eight different mapping populations, where the most constant QTL for grain Zn content across environments was located on chromosome 12 (Swamy et al., 2016), which has synteny to chromosome 5A in wheat (Salse et al., 2009). Therefore, our results indicate potential genomic regions controlling Zn in wheat that can be used in further genetic investigations.

### Identification of Candidate Genes

The gene content of the two genomic regions on chromosomes 3B and 5A harbors many hypothetical and functionally annotated genes or proteins including TFs and transporter proteins (**Supplementary Table S5**). For instance, we found five genes on chromosome 3B related to the MAPK family (**Table 2**) and this gene is well documented in biotic and abiotic stress signaling (Xu and Zhang, 2015). Recently, several publications reported that different MAPK genes play major roles in sugar, nitrogen, phosphate, iron, potassium, or Zn signaling pathways (Lastdrager et al., 2014; Briat et al., 2015; Chardin et al., 2017), which makes them promising candidates for being involved in grain Zn accumulation. The gene annotations of the MEGANTE pipeline showed that one of the MAPK-related genes encoded a vacuolar protein sorting-associated protein. Interestingly, a recent report showed that vacuolar protein sorting-associated protein was identified as one of the candidate genes mediating elevated Zn concentrations in chickpea seeds (Upadhyaya et al., 2016). In the same study, a SWAP/surp domain-containing protein was reported to be linked with seed Zn concentration in chickpea and a SWAP was found in the present study as putative candidate gene on chromosome 3B (**Table 2**).

On chromosome 5A, a homeobox-leucine zipper protein HOX4 that annotated as *TaHDZIP1* was found to be associated with grain Zn concentrations in the used panel. Another regulatory element detected in this genomic region is the putative TF *bHLH76* and it has been reported that bHLH is one of the binding factors of the cis-element G-box which was found in promoter regions of all *TaMTPs* (metal tolerance proteins), which are involved in trace metal homeostasis and have a potential role in cereal grain biofortification with essential micronutrients including Zn (Menguer et al., 2017; Vatansever et al., 2017). Additionally, we found that chromosome 5A harbored a TF belonging to the bZIP (basic-region leucine-zipper) family which have a crucial role in nutrient and Zn homeostasis (Ishimaru et al., 2011; Evens et al., 2017; Cifuentes-Esquivel et al., 2018). In *Arabidopsis thaliana*, the TFs *bZIP19* and *bZIP23* were shown to regulate the adaption to Zn deficiency in roots (Assunção et al., 2010; Inaba et al., 2015). A total of 187 *TabZIP* genes have been identified in wheat (Li et al., 2015) and a specific group of *TabZIP* genes conferred functional complementation of Zn deficiency-hypersensitive such as *bzip19 bzip23* (Evens et al., 2017; Henríquez-Valencia et al., 2018). So far, most functional studies of bZIPs were related to roots or leaves while little information about bZIP-dependent regulatory mechanisms is available for grains. Therefore, novel bZIP genes could play a critical role in improving Zn accumulation in grains. However, this requires further genetic and functional validation. Finally, the FAR1 protein detected on chromosome 5A (**Supplementary Table S5**) and its molecular function based on gene ontology analysis is related to zinc ion binding (**Table 2**), which also makes it a potential candidate gene.

## Conclusion

The present analysis showed the power of the GWAS approach for identifying putative candidate genes for grain Zn accumulation in wheat. This study discovered genetic factors controlling grain Zn accumulation that may establish the basis for further breeding and genetic work in cereals. Two physically anchored chromosomal segments 3B and 5A harbor many putative candidate genes like MAPK and bZIP genes which are proposed as candidates conferring enhanced grain Zn concentrations. Further validation and functional characterization are required to elucidate the role of these genes for Zn homeostasis in wheat.

## Author Contributions

DA performed the data analysis including genome-wide association scan, candidate genes identification, and statistical analysis. KE and NvW participated in Zn concentration measurements. AA helped in manuscript modification and statistical analysis. KP and MR designed the experiment. MR conceived the idea and participated in the interpretation of results. DA and MR wrote the manuscript. All authors read and approved the final manuscript.

## Conflict of Interest Statement

The authors declare that the research was conducted in the absence of any commercial or financial relationships that could be construed as a potential conflict of interest.

## Footnotes

1 http://faostat.fao.org

2 http://www.traitgenetics.com

3 http://www.cerealsdb.uk.net/

4 https://urgi.versailles.inra.fr/WheatMine/begin.do

5 http://www.cerealsdb.uk.net

6 http://www.galaxyproject.org/

7 https://megante.dna.affrc.go.jp/
