# Supplementary figures and images for: Identifying Candidate Genes for Enhancing Grain Zn Concentration in Wheat
Source: Front Plant Sci. 2018 Sep 10;9:1313. doi: 10.3389/fpls.2018.01313 (PMC6143079; doi:10.3389/fpls.2018.01313)

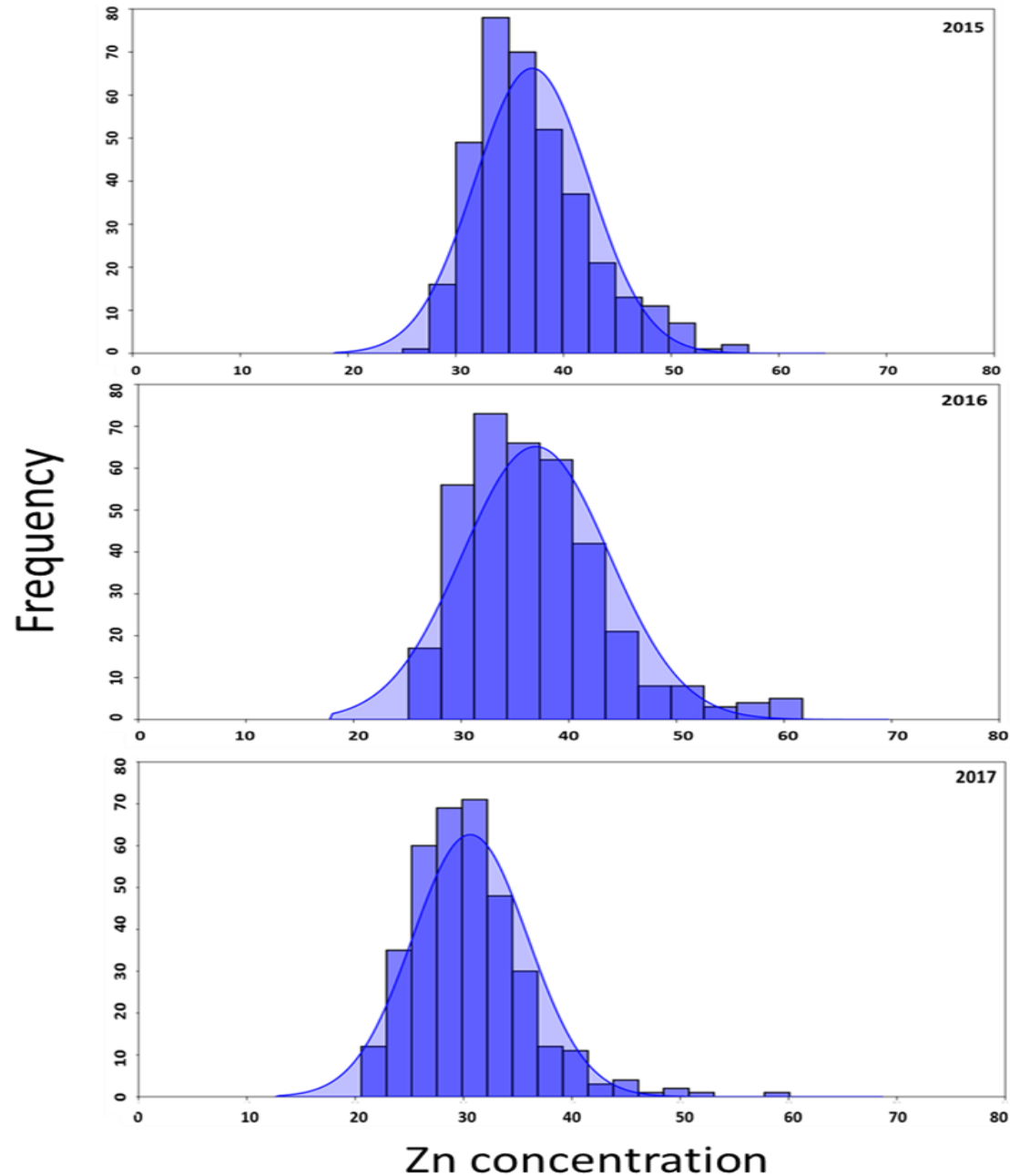

**Figure (S1):** The phenotypic distribution of Zn concentrations in the individual years (2015,2016 and 2017).

Supplement: Supplementary file 1 [file Data_Sheet_1.PDF]

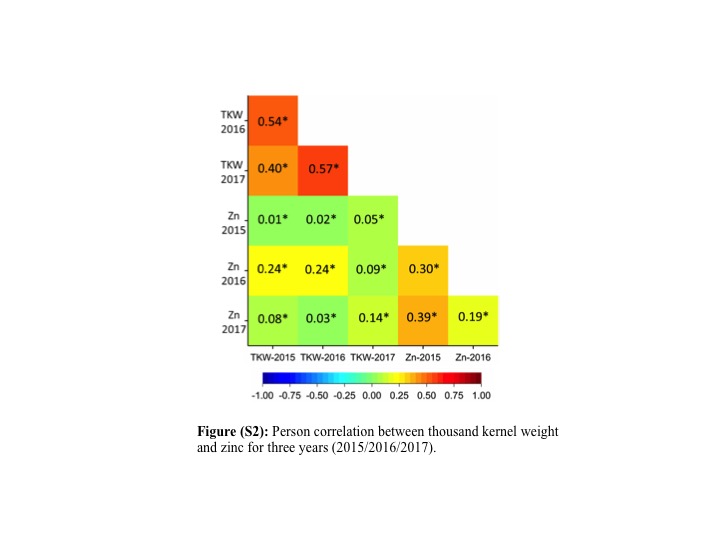

Supplement: Supplementary file 2 [file Image_2.jpg]
