# Supplementary material for: Identifying Candidate Genes for Enhancing Grain Zn Concentration in Wheat
Source: Front Plant Sci. 2018 Sep 10;9:1313. doi: 10.3389/fpls.2018.01313 (PMC6143079; doi:10.3389/fpls.2018.01313)
Supplement: Supplementary file 4 [file Table_2.DOCX]

**Table. S2: Analysis of variance (ANOVA) of Zinc concentration for the European wheat panel (369 genotypes) within three years.**

| **Source of Variation** | **DF** | **SS** | **MS** | **F** | **P** |
| --- | --- | --- | --- | --- | --- |
| **Genotype** | 368 | 19691.401 | 53.509 | 2.171 | <0,001 |
| **Environment** | 2 | 9693.989 | 4846.994 | 196.654 | <0,001 |
| **Residual** | 711 | 17524.287 | 24.647 |  |  |
| **Total** | 1081 | 47036.000 | 43.512 |  |  |

DF = Degrees of freedom

SS = Sum of Squares

MS = Mean of Squares

* P<0.001
